# Effect of Electropulsing Treatment on the Fatigue Crack Growth Behavior of Copper

**DOI:** 10.3390/ma11112168

**Published:** 2018-11-02

**Authors:** Yan Yin, Haibo Chen, Yasuyuki Morita, Yuhki Toku, Yang Ju

**Affiliations:** 1 CAS Key Laboratory of Mechanical Behavior and Design of Materials, Department of Modern Mechanics, University of Technology and Science of China, Hefei 230027, China; yinyan@mail.ustc.edu.cn; 2 Department of Micro-Nano Mechanical Science and Engineering, Nagoya University, Furo-cho, Chikusa-ku, Nagoya 464-8603, Japan; morita@mech.nagoya-u.ac.jp (Y.M.); toku@mech.nagoya-u.ac.jp (Y.T.); ju@mech.nagoya-u.ac.jp (Y.J.)

**Keywords:** fatigue, electropulsing, crack propagation, copper

## Abstract

Crack propagation was quantitatively evaluated to investigate the effect of electropulsing treatment (EPT) on fatigue crack growth of copper specimens. Varying fatigue cycles were obtained under six different load levels. The crack lengths were measured under two load levels to examine the effect of cyclic stress. The microhardness was measured around the vicinity of the crack tip. Furthermore, the fracture surface was observed by scanning electron microscopy. Results show that EPT with electric current density of 150 A/mm^2^ enhances the high-cycle fatigue life, and the effect tends to increase with the decrease in cyclic stress. Vickers microhardness (HV) near the crack tip decreases to normal levels after treatment, and the approaching cracks on two sides can be observed. Local annealing and recrystallization occur around the fatigue crack tip. Accordingly, crack propagation can be delayed, and fatigue life can be prolonged by EPT.

## 1. Introduction

Fatigue is a principal mode of damage which results in metallic equipment failure. Improving long-term durability and reliability of structures has been proven to be essential to prevent fatigue fractures, thereby reducing energy consumption, which is beneficial for the environment. Fine electrical conductivity provides the base of electron wind force by electric treatment in a current-carrying metallic material. Pulsed electric current is effective in improving the mechanical properties of carbon steel and aluminum alloys [1,2]. Zhou et al. [3] indicated that the mechanical properties of cold-worked brass could be improved because of recrystallization caused by electropulsing treatment (EPT). The electropulsing time is very short and smaller grains can be obtained; thus, the mechanical properties of material can be improved. Conrad and Cao et al. [4,5] demonstrated that the fatigue life of copper increased by the treatment of electric current pulse during the rotating bending test. Thermal fatigue behavior of hot work die steel was also positively affected by the pulse current [6]. The EPT is also effective in healing a pre-existing crack. Zhou et al. [7,8] indicated that a quench crack and a through-thickness artificial pre-crack in carbon steel can be partially healed by applying the EPT. Hosoi et al. [9,10] showed that the fatigue crack in stainless steel could be healed by controlling a high-density electric current field. The original microstructure and material performances could remain unchanged after the treatment [11]. A high-density electric current field was formed at the crack tip during EPT, which enhanced fatigue life [12,13,14]. Jung et al. [15,16] examined the effect of pulsed electric current on fatigue cracks of aluminum alloy and found that fatigue crack growth could be affected by different current densities. Investigations for the effect of EPT on fatigue life have been mainly conducted for steel and aluminum alloys, while more information is needed for copper and copper alloys. Copper and copper alloys are widely used in engineering, for example as cooling components and conductor industries, because of their excellent high electrical and thermal conductivities [17]. During the design of synchrotron beam line components, simultaneous enhancement of strength and fatigue resistance are required to facilitate active cooling and mechanical and fatigue properties of copper and copper alloys should be considered [18,19,20]. Although EPT has been proven to cause the recrystallization of cold-worked brass, which increases its mechanical properties [2], the essential effect of this procedure on fatigue crack healing and crack growth propagation of copper has not been observed. Valuable experimental results can be helpful in enhancing the life of fatigued copper components and can aid researchers in improving the design models in their further work to reduce energy consumption.

In the present work, the influence of EPT on fatigue life and crack propagation of copper specimens was examined. Fatigue failure lives were obtained under different load levels to investigate the effect of cyclic stress level. Observations on crack growth behavior were conducted, and the effect of EPT on fatigue crack propagation was quantitatively evaluated by the Paris law. Finally, fracture surfaces were observed to confirm if EPT had changed the fracture mechanism of copper.

## 2. Experimental Details

### 2.1. Specimen

The common copper CuETP (JIS C1100) was used as the experimental material. CuETP is an electrolytic refined copper, which is widely used for electrical and electronic applications. The copper used was commercially annealed and cold-rolled plate, with 5 mm thickness and 99% stated purity. The mechanical properties of the material are tested and presented in Table 1.

**Figure 1:**
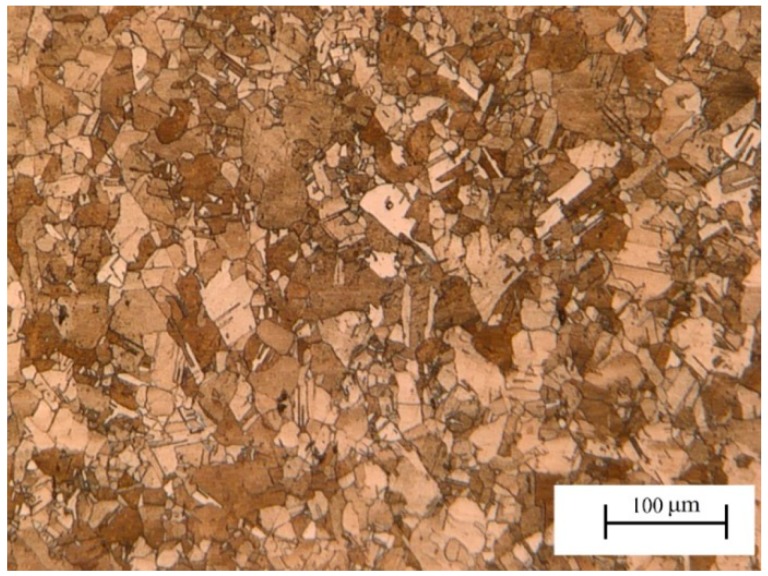
Optical micrograph of the microstructure of C1100 in the original state.

**Figure 2:**
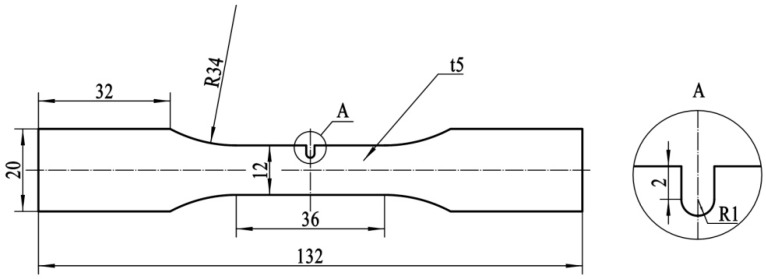
Dimensions of notched specimens.

The mean linear intercept grain size of the material is approximately 16 μm, as shown in Figure 1. Dumbbell-shaped specimens, with a minimum width of 12 mm at the central cross-section, were wire cut along the rolling direction from the copper plate for the fatigue test. A notch was introduced at the center of the edge of one side, as shown in Figure 2. The depth and root radius of the notch were 3 mm and 1 mm, respectively. Thus, the geometric stress concentration factor *K_t_* was 2.26 [21].

The specimen surface was finished with emery papers with grain numbers from 180 to 2000. Finally, the specimen was polished by buffing with alumina powder with 0.05 μm grain diameter to ensure the surface roughness *R_a_* < 0.1 μm.

### 2.2. Fatigue Test and Application of EPT

Reversed push–pull fatigue tests were conducted at room temperature and 10 Hz in a closed-loop, electrohydraulic servo-controlled testing machine (SHIMADAZU 4830, SHIMADAZU Corporation, Nagoya University, Nagoya, Japan). The cyclic stress amplitude applied to the specimen was kept constant. The loading level was controlled in accordance with the nominal stress at the bottom of the U-notch. Six different load levels ranging with amplitude stress of ±50, ±70, ±90, ±111, ±120 and ±138 MPa (21%, 29%, 38%, 46%, 50% and 58% of the ultimate tensile strength) were used for the fatigue tests. Three specimens were used for the fatigue test with and without EPT under each stress loading level. In addition, another specimen was used for the microhardness test.

**Figure 3:**
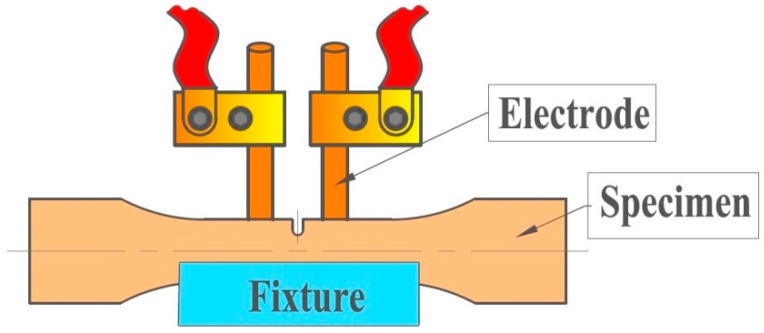
Settings for electropulsing treatment (EPT) of notched copper specimen.

EPT was performed on the specimens by interrupting the fatigue tests at a crack length of approximately 1 mm with a transistor-type power source to investigate the effect of pulsed electric current on fatigue crack propagation. Chromium-copper alloy rods were used as the electrodes. Two electrodes were connected to two sides of the specimen, straddling the notch (Figure 3).

The current density of the applied electric pulse was j = 150 A/mm^2^ with pulsing duration of 0.5 ms. Four current pulses were applied to the fatigued specimen in 5 s. After EPT, the specimen was reset on the fatigue testing machine. The fatigue testing was continued until final failure. The conditions of the electrical stimulation for specimens are shown in Table 2.

### 2.3. Observation of Crack Growth and Fracture Surfaces

The effect of EPT on fatigue crack propagation was investigated at ±50 and ±90 MPa stress levels. The fatigue life of crack propagation is defined as the number of fatigue cycles required for a first observed main surface crack propagating to final failure of the specimen. The crack growth process was monitored with a digital microscope (KEYENCE VH-Z100R, KEYENCE Corporation, Nagoya University, Nagoya, Japan) in real time. After the final failure, the fracture surfaces were observed by the scanning electron microscopy (SEM JEOL JSM-7000FK, JEOL, Ltd., Nagoya University, Nagoya, Japan).

Vickers microhardness (HV) was measured around the vicinity of the crack tip before and after the EPT at the same position of the specimens under load levels of ±50 and ±90 MPa. Six positions in the linear pattern were tested and four spots were tested for each position on both sides of the crack on the surfaces of the specimen. All HV measurements were taken using a standard procedure with 0.2 kg load on a HMV-G SHIMADZU microhardness tester (SHIMADAZU Corporation, Nagoya University, Nagoya, Japan).

## 3. Results

### 3.1. Fatigue Failure Lives

**Figure 4:**
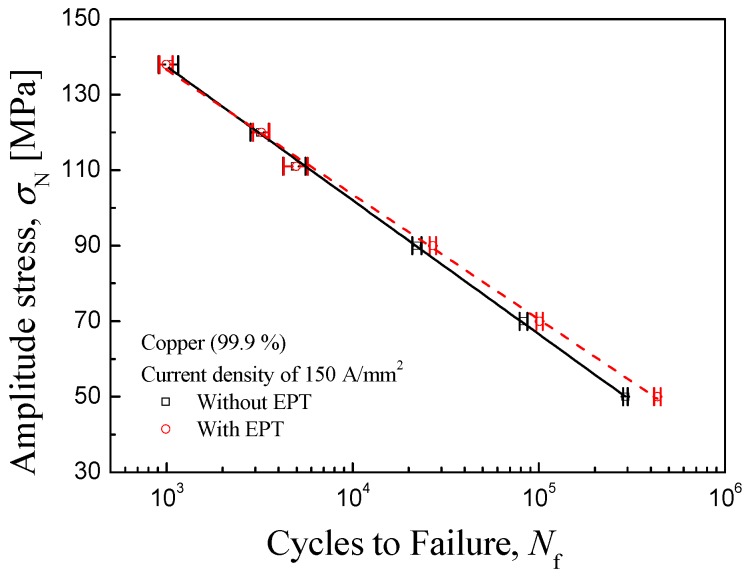
S–N curve of notched specimens with and without current pulsing (j = 150 A/mm^2^, 4 times in 5 s).

The fatigue failure life *N_f_* of the notched specimens was obtained at six different load levels. In all cases, *N_f_* is defined as the cyclic number when a complete separation of the gauge length occurred, and *σ_N_*, which indicates the loading level, represents the nominal cyclic stress amplitude at the bottom of the notch. Figure 4 shows the relationship (S–N curve) between the average number of stress cycles to failure and the nominal stress amplitude. From the figure, it can be seen that fatigue life decreases with the increase in loading stress amplitude, and the *σ_N_*–*N_f_* curves of the notched copper specimens show a uniform Manson–Coffin relationship from low- to high-cycle regions. 

The Manson–Coffin relationship is given by

(1) $${\left(\Delta \sigma /2\right)}^{m}N_{f}=C_{1}$$

where Δ*σ* is the cyclic stress range, *N_f_* is the number of cycles to failure, and *C*_1_ and *m* are material constants. The fitting equations of the Manson–Coffin relationship for the specimens with and without EPT are as follows:

(2) $$\begin{array}{l} {\left(\Delta \sigma_{n}/2\right)}^{5.68}N_{f}=1.95\times 10^{15}\\ {\left(\Delta \sigma_{n}/2\right)}^{6.09}N_{f}=1.43\times 10^{16} \end{array}$$

The S–N curve after EPT is uniformly above that without EPT. The material constant *m* increases from 5.68 to 6.09, which is consistent with the reduced slope after applying electropulsing in Figure 4. For the specimens under a load amplitude of more than ±90 MPa, fatigue lives were nearly the same with or without EPT. At the lowest stress of ±50 MPa, the fatigue life of the specimen with EPT was approximately 1.5 times longer than that of the specimen without EPT. The positive influence on fatigue life by EPT tends to decrease with the increase in stress *σ_N_*, as is reported by Conrad [4].

### 3.2. Microhardness Test

**Figure 5:**
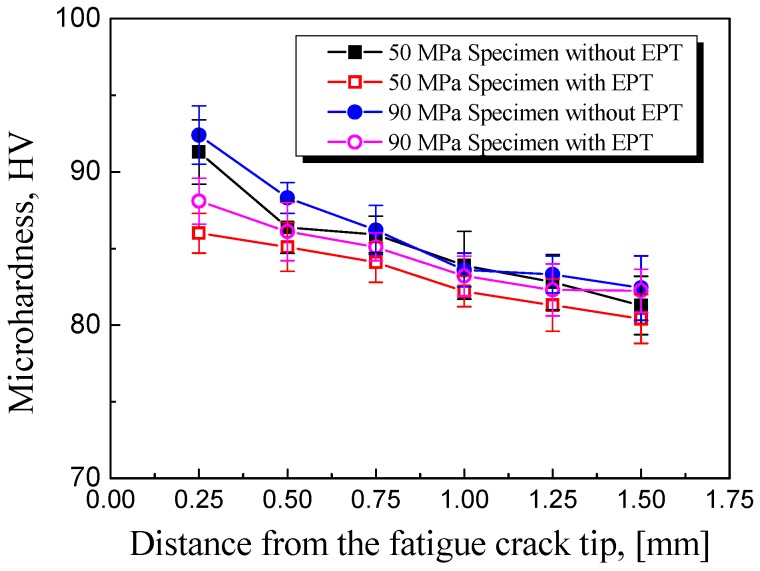
Results of Vickers microhardness (HV) measurements for specimens with and without EPT.

Microhardness HV measurements were conducted on the same fatigued specimen before and after EPT. Figure 5 shows the relationship between HV and the distance from the edge of the crack tip.

Figure 5 presents the HV results before and after EPT. The square symbols are the results of specimens under stress level ±50 MPa while the circular symbols are the results of stress level ±90 MPa.

As shown in Figure 5, the HV near the crack tip was higher than that far from the crack tip because of the plastic deformation after cyclic loading. HV decreased with the increase of distance to the crack and became almost the same as the average hardness of the as-received material (82.4 HV) at distance 1.5 mm. The decrease range of copper is 32 HV after traditional annealing for 1 h under 300 °C [22]. After applying EPT, the previously higher HV around the fatigue crack decreased close to the nominal hardness of the original material and the decrease range of about 5 HV at distance 0.25 mm is substantial due to electropulsing. This result demonstrated that the plastic strain caused by cyclic loading had been partially released, which was related to the recrystallization of the plastic zone near the fatigue crack.

### 3.3. Fatigue Crack Growth Behavior

**Figure 6:**
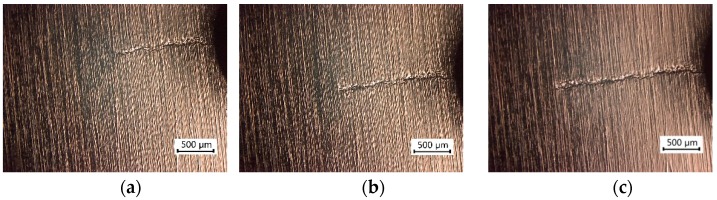
Crack growth from the notch of the specimen with EPT (±50 MPa, *N_f_* = 433,896). (**a**) *N/N_f_* = 60%; (**b**) *N/N_f_* = 66%; (**c**) *N/N_f_* = 73%.

**Figure 7:**
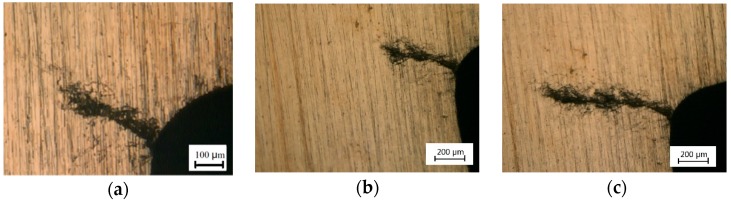
Crack growth from the notch of the specimen with EPT (±90 MPa, *N_f_* = 18,898). (**a**) *N/N_f_* = 61%; (**b**) *N/N_f_* = 66%; (**c**) *N/N_f_* = 74%.

Figure 6 and Figure 7 show the fatigue crack growth behavior of two specimens with EPT under cyclic stress amplitudes of ±50 and ±90 MPa, respectively. The fatigue cracks initiated at the bottom of the U-notch and propagated perpendicular to the stress loading direction. Evidently, the fatigue crack of the specimen under ±90 MPa loading level was wider than that under ±50 MPa. A wide crack could be identified with a large plastic zone near the fatigue crack.

The length of the main crack observed on the surface was measured according to the digital microscope photo. Crack length is measured from the bottom of the U-notch to the fatigue crack tip. The valid crack growth rate was used after a microcrack had grown to a length approximately three times that of the average grain size [23], which was about 50 μm for pure cropper, to prevent uncertainty associated with the definition of crack propagation life. Crack growth behavior was evaluated quantitatively by the Paris law, whereas the stress intensity factor (*K*) was calculated by finite element analysis using the commercial software ABAQUS (version 6.14) to observe the effect of EPT on crack growth.

**Figure 8:**
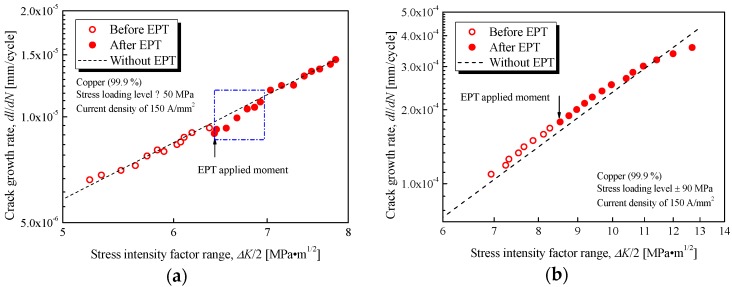
Fatigue crack growth rate as a function of the stress intensity factor range for specimens with and without EPT: (**a**) ±50 MPa loading level; (**b**) ±90 MPa loading level.

Figure 8a,b show the relationship between fatigue crack growth rate and stress intensity factor range obtained by applying stress levels of ±50 and ±90 MPa, respectively. The dashed line indicates the result for the untreated specimen under the same stress loading level. The open symbols show the crack growth behavior before EPT, and the solid symbols show the crack growth behavior after applying EPT and resetting for the fatigue test. No effect was observed on crack growth by removing a specimen from the testing machine and resetting it again [9]. The difference between the open and solid symbols is mainly caused by EPT.

The result with a ±50 MPa stress loading level shows that the crack growth rate decreased only after electrical treatment. After the application of EPT (as shown in the Figure 8a by the arrow), the solid symbols dropped and were all below the dashed line until the half stress intensity factor range reached about 7 MPa·m^1/2^. The crack growth rate decreased in the square region marked in the figure. The distance between the solid symbols and dashed line is larger than that caused by error scatter, especially for the subsequent several thousands of loading cycles after EPT. The retardation of crack propagation was sustained for several thousands of loading cycles and then returned to the standard crack growth rate. 

However, the crack growth rate nearly remained unchanged for the specimen under a ±90 MPa stress loading level, as shown in Figure 8b. A large stress intensity factor was induced by high loading stress. Moreover, the fatigue crack was wide for a large plastic deformation region; thus, the concentration of the electric current reduced at the fatigue crack tip because of its large crack root radius. In addition, the decreasing slope of the curve above about 12 MPa·m^1/2^ in Figure 8b may be caused by pronounced plastification in front of the crack tip, but not related to the application of EPT.

As a result, the fatigue crack growth behavior was influenced by the application of proper electric current. Furthermore, the fatigue life would increase by the delay effect of the electric current treatment on fatigue crack propagation.

EPT has a minimal influence on crack growth and fatigue life for the specimens under ±90 MPa loading levels. Hence, the subsequent results focus on the specimens under ±50 MPa loading level in this study

### 3.4. Observation of the Fatigue Crack Tip

**Figure 9:**
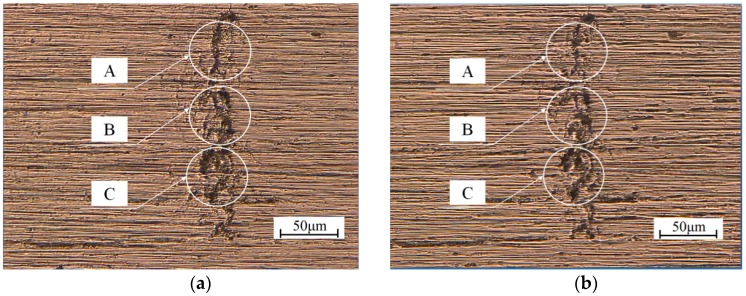
Crack tip of the fatigue crack in the specimen under the ±50 MPa loading level: (**a**) before EPT; (**b**) after EPT.

Figure 9a,b show the digital microscope photographs of the fatigue crack tip before and after EPT for the specimen under a ±50 MPa loading level. Although the crack was not entirely healed after EPT, several parts of the crack became narrower after EPT, especially for Region A in Figure 9b in comparison with that in Figure 9a. The width of the main crack became narrower in Region A due to the electrical treatment. Similar phenomena can be seen from Regions B and C in Figure 9. The two surfaces of the main crack came closer to each other and the fatigue crack was partly healed after EPT.

### 3.5. Fracture Surface

**Figure 10:**
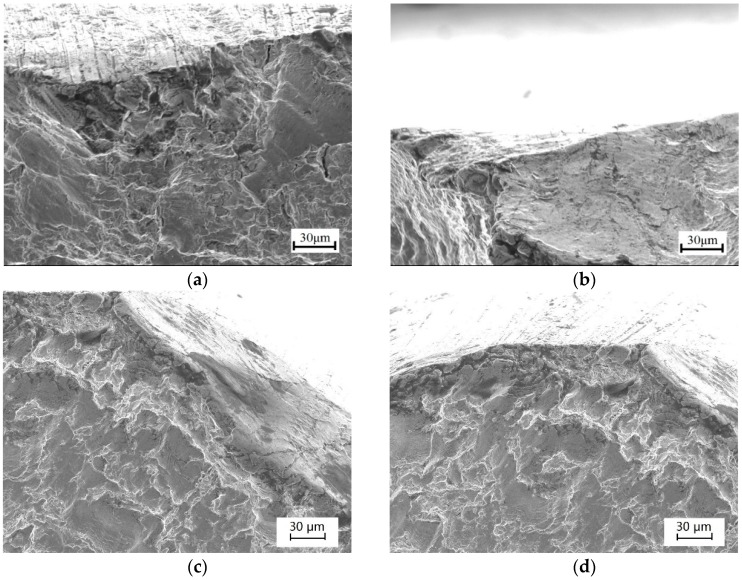
Surface crack ridges of the specimens taken by SEM: (**a**) ±50 MPa loading level without EPT; (**b**) ±50 MPa loading level with EPT; (**c**) ±90 MPa loading level without EPT; (**d**) ±90 MPa loading level with EPT.

Figure 10a–d show the fracture topography near the specimen surface after the fatigue tests. Figure 10a,c are the SEM micrographs of the fracture surfaces in as-received specimens without EPT. The fatigue crack initiated at the bottom of the U-notch and propagated with cyclic slip deformation along both directions in width and thickness. The rough morphology on the surface of the crack revealed the plastic deformation of the material. Figure 10b,d show the fracture surfaces of the specimen after EPT by applying pulsed electric current with 150 A/mm^2^ density. No evidence of melting could be observed near the crack surface, which differs from results reported for an aluminum alloy [15]. The rise in temperature caused by EPT was insufficient for the melting of copper, and only recrystallization occurred. Recrystallization induced fine grains near the fatigue crack tip and the release of plastic strain. Therefore, the fatigue life increased with EPT by the recrystallization of copper, which corresponds to the HV test result.

**Figure 11:**
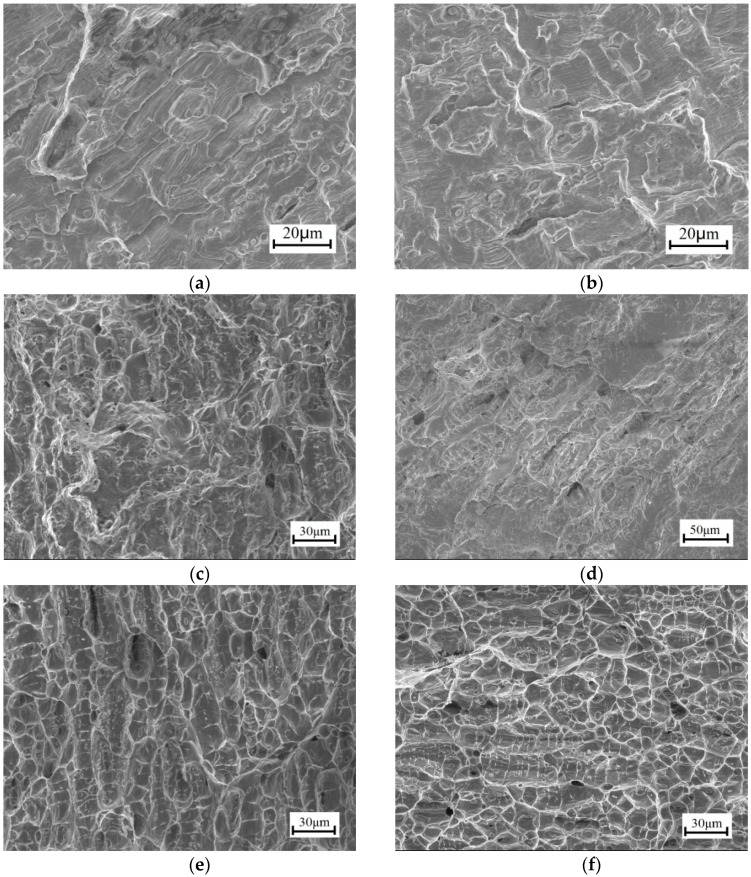
Scanning electron micrographs of the fatigued copper specimen under a ±50 MPa loading level: (**a**) Crack propagation region without EPT; (**b**) crack propagation region with EPT; (**c**) unstable growth crack growth region without EPT; (**d**) unstable crack growth region with EPT; (**e**) final fracture region without EPT; (**f**) final fracture region with EPT.

Fracture surfaces of as-received and EPT specimens under a ±50 MPa loading level were observed by SEM (Figure 11). A comprehensive examination of crack growth revealed the microscopic crack initiation at the bottom of the notch, followed by a stable microscopic crack growth. In the region of stable microscopic crack propagation, the cracks traversed along the slip planes within each grain. Although the notch caused stress concentration in the specimens, the considerable cycles spent for growth of the microscopic and macroscopic cracks through this region covered a significant percentage of the total fatigue life. After the crack propagation, unstable crack growth progressed before the final fracture occurred. Thus, the crack propagation, unstable crack growth, and final fracture regions were identified to discover the fracture features of copper specimens.

In the crack propagation region (Figure 11a,b), fatigue striations spreading uniformly distributed through the fracture surface, and numerous fine microscopic and macroscopic cracks meandering through the matrix and tearing ridges formed along the direction of the main fatigue crack. The average width of fatigue striations in Figure 11b became narrow compared with those in Figure 11a, thereby indicating that crack propagation rates were delayed by EPT application. In the region of unstable crack growth (Figure 11c,d), few of the microscopic voids coalesced, and the halves of these voids were the dimples visible on the fracture surface. The fracture appearance in the region of unstable crack growth comprised pronounced cracking along the grain boundaries and a distribution of dimples adjacent to the grain boundary. At the microscopic level, fatigued copper revealed a rough morphology in the final fracture region, and the isolated pockets of shallow dimples on the transgranular fracture surface (Figure 11e,f) indicated the ductile nature of failure of the drawn microstructure. 

For the two specimens, minimal changes in microscopic fracture features were observed for the unstable crack growth and final fracture regions. The fracture mechanism of copper did not change after EPT. Thus, the mechanical properties of the main material remained unchanged after treatment.

## 4. Discussion

Joule heating is caused by EPT for metallic materials. Considering that the electric treatment is conducted in an extremely short time, it can be regarded as an adiabatic course. Zhou et al. [3] reported that temperature rise could reach 612 °C, and recrystallization microstructure was produced in cold-worked brass sheet by EPT with extremely high current density of 17 kA/mm^2^ in 0.13 ms.

**Figure 12:**
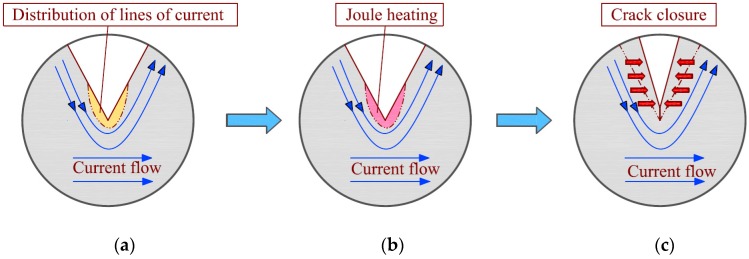
Representation of the EPT application: (**a**) Concentration of electric current field around a crack tip; (**b**) local Joule heating caused by electric current field around a crack tip; (**c**) crack closure resulting from thermal expansion and thermal compressive stress.

For the specimen with a pre-crack or fatigue crack, the crack can be healed to a certain degree, and the fatigue life can be prolonged by EPT with low current densities [7,8,9,10]. The principle of the effect of EPT on a fatigue crack is represented schematically in Figure 12. When the electric current is applied across a crack, it flows along the crack because of the electrical resistance on the crack surfaces. Therefore, a high-density electric current field forms around the crack tip, where local heating can concentrate. Thus, local annealing, recrystallization, and even melting can occur by Joule heating. The tensile residual stress around the crack tip is finally released by local annealing [24]. Thermal compressive stress occurs due to the rapid local thermal expansion at the crack tip and its vicinity. The surfaces of the main crack will then become close to each other by thermal expansion. The crack closure reduces the driving force for crack propagation because of the decrease of the crack opening displacement. 

Moreover, part of the crack will melt and heal if the local temperature reaches the melting point of the material. A fatigue crack in austenitic stainless steel has closed and bridged after EPT (maximum current density of approximately 356 A/mm^2^) because of partial melting that occurs between the crack surfaces [9]. Jung et al. [15,16] investigated the local melting structure from the fracture surface after EPT with a current density of 90 A/mm^2^ that was introduced to a fatigued aluminum alloy specimen. In the present study, copper specimens are used for the tests; however, no evidence of melting has been observed due to their high electric conductivity. 

For the specimens under a ±50 MPa loading level, the decrease in microhardness HV shows that local recrystallization has occurred around the crack tip, and the crack surfaces come closer to each other, as shown in Figure 9. Electropulsing can increase the nucleation rate in the early stage of recrystallization and retard the subsequent rate of grain growth. Fine grains have formed uniformly with EPT, resulting in improved mechanical properties. Retardation of crack growth is caused by the existence of the fine grains. However, the retardation on crack propagation is only temporary. The growth rate will return to its standard value after several thousands of loading cycles when the crack propagates out of the recrystallization region, as shown in Figure 8a. Meanwhile, the tensile residual stress promotes fatigue crack propagation. The residual stress is released by local annealing with Joule heating at the crack tip, which also has an influence on the resistance of crack growth. Therefore, fatigue life is prolonged by crack closure and local annealing in macroscopic and microscopic viewpoints, respectively.

For the specimens under a ±90 MPa loading level, although microhardness HV also decreases after EPT, the pulsing current has minimal effect on the crack propagation and entire fatigue life. The cracks were wider and the crack root radii were larger than those of specimens under the lower ±50 MPa loading level, as shown in Figure 6 and Figure 7, respectively. The current density distribution around the crack tip is related to the sharpness of the crack tip, and weak current concentration factor is induced by a large root radius of the crack tip [25]. Although local annealing around the crack tip has also occurred, a spare space exists between the crack surfaces for thermal expansion. Thus, thermal compressive stress cannot sufficiently cause crack closure in this situation. Moreover, plastic deformation due to fine ductility leads to rough surfaces for the crack. As shown in Figure 10, the rougher crack surfaces are, the more the adhesion between the crack surfaces is prevented. The electric current density applied in the present test is extremely low for the specimens under a ±90 MPa loading level. Hence, the crack nearly remains unchanged. 

A similar result also indicated that EPT has a considerable effect on a high-cycle fatigued specimen [4]. The crack growth rates are remarkably decreased due to the effect of pulsed electric current for the crack specimen with a low stress intensity factor [15,26]. In essence, the effect of loading level on crack healing is related to plastic strain and deformation degree. Narrow crack and restorable deformation are induced under low loading levels. Plastic deformation with a wide crack is induced under high loading levels. In this study, the high-cycle fatigue process tends to be sensitive to EPT with 150 A/mm^2^ for 0.5 ms. By contrast, the influence on crack propagation and fatigue life is ineffective for the low-cycle fatigue process. Applying the electric current with appropriate density is essential. Additional treatment, such as surface-activated pre-coating, is also preferred for closing and healing a specific crack [27,28].

In summary, the effect of EPT on fatigue life and crack growth for ductile material copper is conducted in this work. The results indicate that the crack growth rate has been delayed and the fatigue life is enhanced after applying EPT. The advantage of the EPT method to enhanced fatigue life is shown as two aspects: Firstly, EPT was done in a very short time, which is very convenient and energy saving comparing to traditional annealing. Secondly, the electropulsing has a selective effect. During the process of EPT, with high current density passing through a metal sample with a fatigue crack or other badly injured part, the temperature increase is significant and the effect of electropulsing is strong due to the big regional resistivity and the strong detour of electric current in the local area around the crack. However, in the area without an injured part, the effect of electropulsing is weaker. Local recrystallization has occurred around the fatigue crack tip after EPT and the plastic strain caused by cyclic loading has been partially released. Therefore, EPT is an effective method for healing the damage while the main material remains unchanged.

This research focused on the influence of EPT on fatigue life and fatigue crack propagation. The plate specimens with a U-notch at one side were used for the experiment to make sure the crack initiated and propagation occurred at the bottom of the notch. A digital microscope was used for the observation and measurement of the crack length. The positive influence of EPT on fatigue behavior of copper has been confirmed, and it would be wise to attempt to apply this method to improve the lifetime of complex structures, even the actual components in engineering. However, observation and crack length measurement by the digital microscope may not be so convenient for the actual components with complex geometry in engineering. Progressive methods of crack length measurement, for example Digital Imaging Correlation (DIC), X-ray Tomography and Thermoelastic Stress Analysis [29,30,31,32,33,34], will be considered for complex specimens and structural components in our future work. In addition, the fatigue characteristics and fracture mechanism of the main material remain unchanged because of the selective effect of EPT. As the microstructure cannot be quantitatively correlated to the material properties by the present SEM observation [35], research on the effect of EPT on dislocations, slip bands and grain boundary will be conducted in our follow-up research with the Transmission Electron Microscope (TEM) and the Electron Contrast Channeling Imaging in a field emission SEM. The effect of electropulsing on fatigue crack initiation of copper will also be discussed in our future work.

## 5. Conclusions

The effect of EPT on crack propagation of copper specimens under a reversed pull–push fatigue test was examined in this study. On the basis of the detailed investigation on the influence of EPT on fatigue life, crack growth progress, microhardness around the crack tip, and fracture surface of copper, the following key observations are established:

1. The S–N curves of the specimens with and without EPT followed the Manson–Coffin relationship and exhibited a single slope behavior for the variation of cyclic stress amplitude from low- to high-cycle regions.
2. The EPT with current density of 150 A/mm^2^ enhanced high-cycle fatigue life. This enhanced effect tends to increase with the decrease in cyclic stress. However, the electric current hardly affects the low-cycle fatigue life of copper specimens.
3. The measurement of microhardness revealed evidence on releasing of cyclic hardening through EPT, that is, the local recrystallization occurred around the tip of the fatigue crack. Crack propagation was delayed during the first few thousand cycles after the treatment, then regained the initial growth curve to failure.
4. In the present test, no evidence of melting or blunting was observed around the crack tip on the fracture surfaces by SEM. Thus, the increase of fatigue life by EPT was mainly caused by local annealing and recrystallization around the fatigue crack tip.
5. From a microscopic viewpoint, a minimal difference exists between the propagation and the final fracture regions on the fracture surfaces of specimens with and without EPT. Therefore, the fracture mechanism remains unchanged after the EPT.

## Figures and Tables

**Table 1:** Mechanical properties of C1100.

Yield Strength at 0.2% Offset (MPa)	Tensile Strength (MPa)	Poisson’s Ratio	Young’s Modulus (GPa)	Elongation (%)	Hardness (HV)
180	240	0.33	118	28	82.4

**Table 2:** EPT conditions applied in fatigued specimens.

Stress Level [MPa]	±50	±90
Current density [A/mm^2^]	150	150
Pulsed duration [ms]	0.5	0.5
Number of times of current pulse in 5 s	4	4
